# Minimization of Cancellation Effect with Nisin During Bipolar Nanosecond Electrochemotherapy

**DOI:** 10.3390/ijms27104523

**Published:** 2026-05-18

**Authors:** Veronika Malyško, Aušra Nemeikaitė-Čėnienė, Olga Michel, Arnoldas Morozas, Zofia Łapińska, Eglė Mickevičiūtė-Zinkuvienė, Paulina Malakauskaitė, Augustinas Želvys, Barbora Lekešytė, Justinas Ivaška, Julita Kulbacka, Vitalij Novickij

**Affiliations:** 1 Faculty of Electronics, Vilnius Gediminas Technical University, LT-10223 Vilnius, Lithuania; egle.mickeviciute@imcentras.lt (E.M.-Z.); paulina.malakauskaite@imcentras.lt (P.M.); augustinas.zelvys@imcentras.lt (A.Ž.); barbora.lekesyte@imcentras.lt (B.L.); 2 Department of Immunology and Bioelectrochemistry, State Research Institute Centre of Innovative Medicine, LT-08406 Vilnius, Lithuania; ausra.ceniene@imcentras.lt (A.N.-Č.); olga.michel@umw.edu.pl (O.M.); zofia.lapinska@student.umw.edu.pl (Z.Ł.); julita.kulbacka@umw.edu.pl (J.K.); 3 Department of Molecular and Cellular Biology, Faculty of Pharmacy, Wroclaw Medical University, 50-556 Wroclaw, Poland; 4 Department of Personalized Medicine, State Research Institute Centre of Innovative Medicine, LT-08406 Vilnius, Lithuania; arnoldas.morozas@santa.lt (A.M.); justinas.ivaska@santa.lt (J.I.)

**Keywords:** bipolar cancellation effect, nisin, pulsed electric field, nanosecond, high-frequency, bleomycin, electrochemotherapy, permeabilization, in vitro, viability

## Abstract

Bipolar cancellation (BPC) is an efficiency-limiting phenomenon in bipolar nanosecond pulsed electric field (nsPEF) exposures, in which the second, opposite-polarity phase reduces or partially reverses the electroporation induced by the first phase. Nisin, a cationic antibiotic peptide, has been reported to interact with lipid membranes in bacterial systems and artificial bilayer models, where it may contribute to membrane destabilization and increased permeability during pulsed electric field exposure. This study investigated whether nisin may enhance the efficacy of bleomycin electrochemotherapy (ECT) in the presence of bipolar nanosecond pulses, which are typically associated with pronounced BPC effects. Pulsed electric field (PEF) parameters and drug concentrations were selected based on preliminary viability and Yo-Pro-1 uptake experiments in CLS-354 human squamous cell carcinoma cells. To evaluate the effect of nisin, cell viability and membrane permeabilization were assessed following exposure to 300 ns pulses across a range of bipolar PEF protocols, with or without nisin, while identical unipolar pulses were used for comparison. Nisin (50 µg/mL) increased membrane permeabilization across the tested range of field amplitudes (9–15 kV/cm) and burst repetition frequencies (0.1–1.66 MHz). The presence of nisin was also associated with increased efficacy of bleomycin-based ECT under both unipolar and symmetrical bipolar PEF conditions. Under the optimized parameters tested (13 kV/cm; 150 pulses of 300 ns at 1.66 MHz), bipolar nsPEFs in combination with nisin reached levels of efficacy comparable to those observed with unipolar waveforms, suggesting a potential attenuation of bipolar cancellation effects.

## 1. Introduction

Theoretical and experimental studies have shown that exposure of cells to pulsed electric fields (PEFs) increases the induced transmembrane potential (TMP). When this potential exceeds a critical threshold, molecular rearrangements occur within the lipid bilayer, resulting in the formation of hydrophilic pores through a process known as electroporation (EP) [1,2,3,4]. EP-based procedures found application across diverse fields from food processing [5,6] to healthcare, with applications including wound healing [7,8], scar prevention [9], cardiac ablation [10], gene electrotransfer [11,12] or cancer treatment [13,14]. The latter PEF applications are particularly dependent on their parameters—high intensity electric fields cause irreversible cell damage and are therefore used for tissue ablation [15,16]. By contrast, lower PEF intensities produce transient pores that can last for seconds or even minutes after pulse application [17,18]. This process is more commonly used to deliver therapeutic agents, especially chemotherapeutics, and is referred to as electrochemotherapy (ECT) [19,20,21]. The effectiveness of ECT depends both on the intrinsic efficacy of the drug and on its efficient delivery, which is achieved through appropriately selected PEF parameters. The most clinically established protocol, ESOPE, uses microsecond-duration unipolar pulses [22]. Despite promising ESOPE outcomes across many cancer types [23,24], several ECT-associated side effects have been identified, including pain [25], muscle contractions [26,27], non-uniform treatment due to conductivity variation within tissues [28,29], and thermal damage [30,31]. These problems may be addressed by employing shorter electric pulses, using either unipolar [32,33] or bipolar [34,35] waveforms. By doubling the pulse frequency component, biphasic pulses help reduce muscle contractions and pain and, perhaps most importantly, diminish conductivity variation across tissue layers—especially the highly resistive stratum corneum—yielding a more uniform treatment [36,37]. Unfortunately, the application of bipolar pulses entails a phenomenon known as the bipolar cancellation (BPC) effect. BPC is the loss or reduction in electroporation effectiveness that stems from the combination of pulses of equal, opposite-polarity phases. As the second phase drives charge in the reverse direction, it may partially or entirely negate the effects evoked by the first phase by reducing the transmembrane potential, which leads to nanopore closure and weakening of the cellular responses to PEFs [38,39,40]. This process is prominent especially for short pulses and worsens with higher pulse repetition frequencies [41,42]. To this day, several strategies have been employed to counteract the BPC phenomenon—these include generating pulse asymmetry in terms of amplitude and duration [43] or the introduction of a delay between phases of opposite polarity [44,45,46]. Another promising, however unexplored route to mitigate BPC would be the use of external agents supporting the preservation of conductive pores during the opposite phase. This view is supported by studies showing that electroporation can be greatly boosted by pore-stabilizing agents [47,48], one of them being nisin [49]. Nisin, a naturally occurring antimicrobial peptide, is primarily known for its potent activity against Gram-positive bacteria [50] and is widely used in the food industry [51]. In addition to its antimicrobial effects, nisin has been identified as a potent adjuvant, enhancing the cytotoxicity of chemotherapeutic agents against various cancer cell lines [52], also through apoptosis [53]. Nisin’s structure enables it to adopt a conformation that facilitates protein aggregation at pore edges, reducing the line tension [54]. This may lead to increased membrane permeability after PEF application as demonstrated by studies on liposomes [49] and bacteria [55,56]. This study aimed to evaluate the effect of nisin on enhancing the efficacy of symmetrical bipolar nanosecond pulsed electric fields (nsPEFs) by reducing bipolar cancellation.

The research focused on 100 and 300 ns bipolar pulses, and the effects of nisin on cell membrane permeabilization and bleomycin-based ECT were characterized. CLS-354 was used as a cell model. Partial mitigation of BPC with nisin could pave the way for the clinical use of bipolar nsPEFs and broaden the ECT drug portfolio—either as a standalone agent or as an adjuvant.

## 2. Results

### 2.1. Cell Membrane Permeabilization Assay

**Figure 1:**
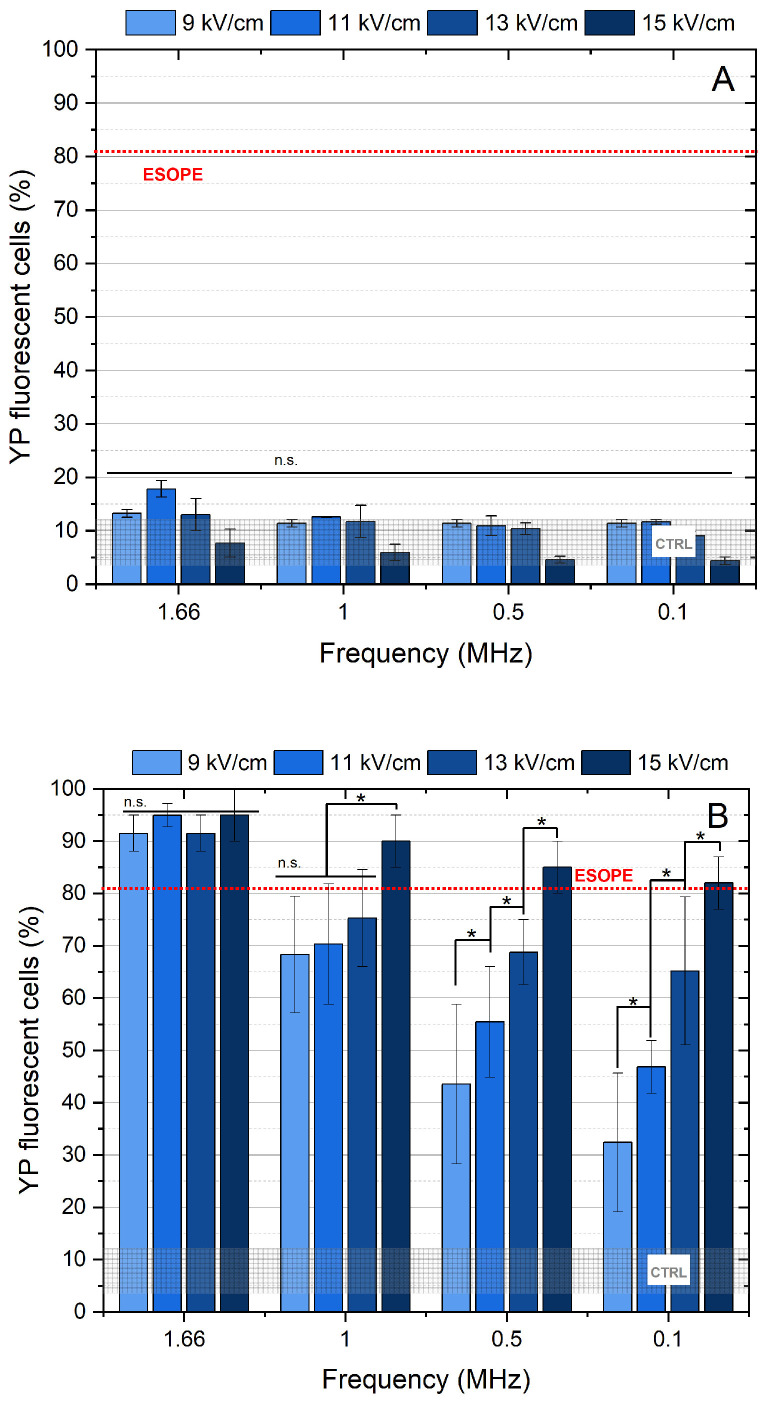
The relationship between cell membrane permeabilization and electric field intensity, pulse duration, and applied burst frequency using unipolar PEFs without the addition of nisin, demonstrating the (**A**) effect of PEF protocol using 100 ns duration pulses and (**B**) effect of PEF protocol using 300 ns duration pulses. ESOPE refers to 1.2 kV/cm × 100 µs × 8, 1 Hz protocol. Data presented as average ± SD. One-way ANOVA: Asterisk (*) *p* < 0.05, n.s.—*p* > 0.05. CTRL indicated the untreated control samples.

To establish the most efficient protocols for cell membrane permeabilization, we first tested the efficacy of unipolar 100 and 300 ns pulse sequences, varying the pulse amplitudes and pulse repetition frequency from 1.66 to 0.1 MHz. The results are summarized in Figure 1. The results indicate that the PEF modality employing 100 ns pulses did not significantly increase YP uptake in the cells, regardless of pulse repetition frequency or amplitude (Figure 1A).

On the contrary, PEF protocols featuring 300 ns pulse durations allowed for achieving higher cell membrane permeabilization rates, which were clearly dependent on burst frequency and amplitude (Figure 1B). It is evident that a reduction in pulse repetition frequency triggers up to several-fold lower cell membrane permeabilization rate (i.e., at 9 kV/cm, permeabilization reached a plateau, but at 0.1 MHz, only ~30% of cells were permeabilized). It is shown that, at 1.66 MHz, the permeabilization was saturated for the whole range of amplitudes involved in the study, indicating that pulse repetition frequency can be used as an additional degree of freedom for the derivation of effective PEF protocols. The ESOPE protocol (1.2 kV/cm × 100 µs × 8, 1 Hz) was used as a positive control/reference and induced permeabilization in >80% of cells. For further ECT experiments, protocols achieving ESOPE-equivalent or greater permeabilization are required.

**Figure 2:**
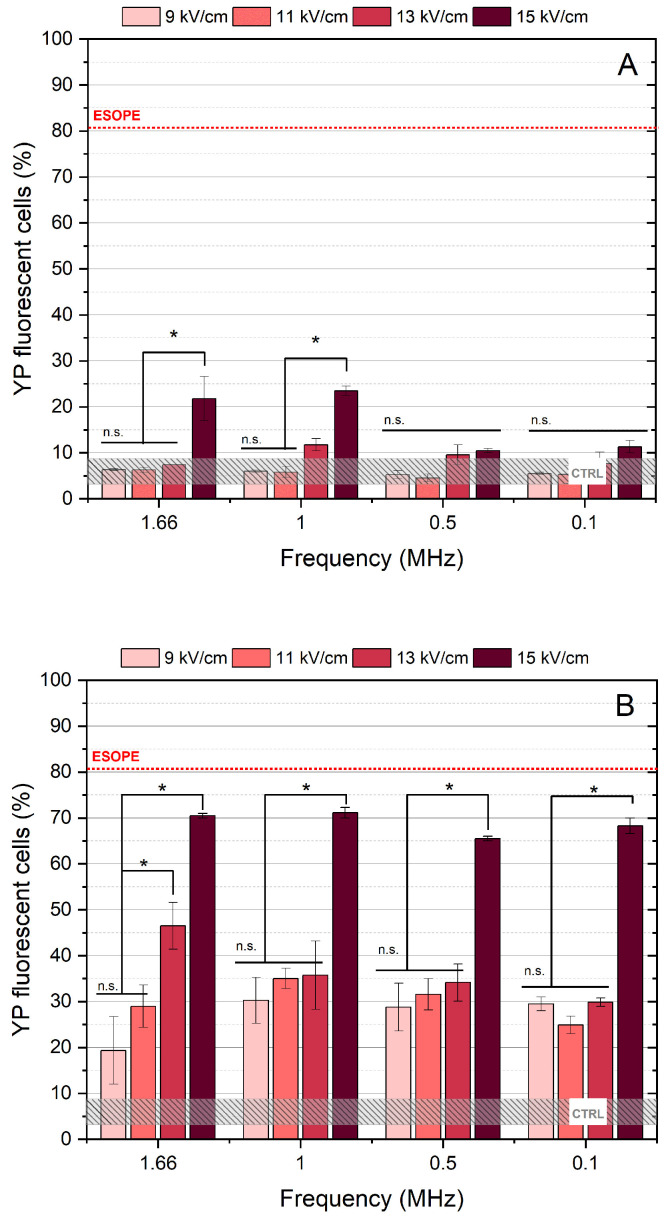
The relationship between cell membrane duration of 300 ns (↑300 ns + 0 ns + ↓300 ns + delay ns), where delay was adjusted with regard to pulse repetition frequency. (**A**) Samples without nisin. (**B**) Samples treated with nisin. ESOPE refers to 1.2 kV/cm × 100 µs × 8, 1 Hz protocol. Data presented as average ± SD. One-way ANOVA: * *p* < 0.05, n.s.—*p* > 0.05. CTRL corresponds to the untreated control samples.

For electroporation using bipolar pulses, the 100 ns duration was not used due to its inefficiency within the studied range of parameters (refer to Figure 1). Therefore, 9–15 kV/cm PEF with pulse durations of 300 ns for both the positive (↑300 ns) and negative (↓300 ns) phases were used. The obtained results are presented in Figure 2.

In the absence of nisin, cell permeabilization was negligible, despite the double total burst energy (two phases of 300 ns pulses). Only the 15 kV/cm protocol produced a statistically significant increase in electroporation rate (~20%), which is insufficient for ECT and still confirms a strong BPC effect. Since bipolar pulses were expected to trigger a strong BPC phenomenon (Figure 2A), nisin was introduced into the study.

However, when nisin was included, the same pulsing protocols resulted in a statistically significant increase in permeabilization rates (Figure 2B). It was still evident that nisin could not fully compensate for the BPC effects; however, it enabled permeabilization rates exceeding 50%, representing a several-fold increase. The effect of pulse repetition frequency was minimal compared with that observed for unipolar pulses.

Based on the results of the permeabilization assay, the 13 kV/cm protocols delivered at 1.66 MHz repetition frequency (both unipolar and bipolar) were used for subsequent ECT experiments. This field strength was selected to investigate nisin effects under conditions where the BPC phenomenon remained strongly pronounced rather than being partially mitigated by higher PEF amplitudes.

### 2.2. ECT-Compatible BLM Concentration

**Figure 3:**
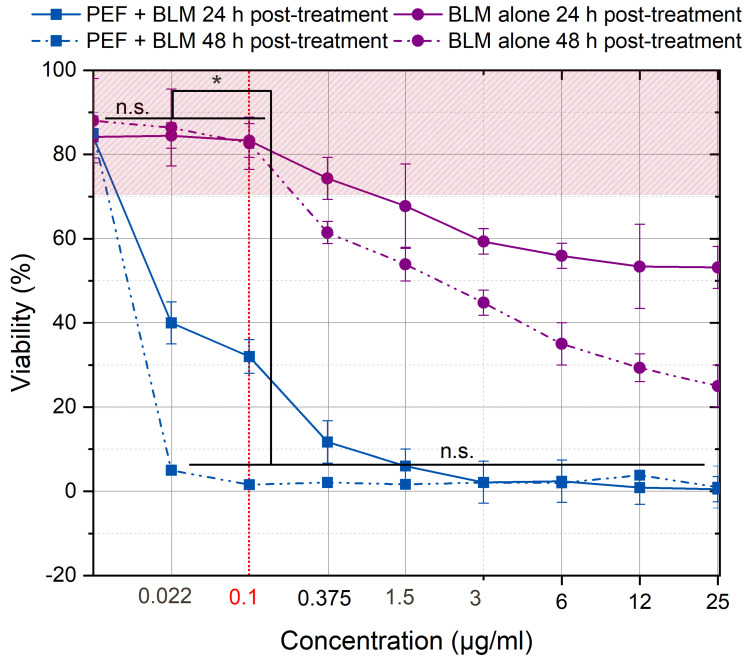
Dependence of ECT efficiency on drug concentration evaluated 24 h (solid line) and 48 h (dashed line) post-treatment in the presence and absence of PEF (ESOPE protocol: 1.2 kV/cm × 100 µs × 8, 1 Hz). The highlighted area represents cell viability >75%. Data presented as average ± SD. One-way ANOVA: Asterisk (*) *p* < 0.05, n.s.—*p* > 0.05.

An ECT-compatible concentration of bleomycin (0–25 µg/mL) was determined based on 24 and 48 h viability assays in the presence and absence of PEF (Figure 3). The experiments were performed using the ESOPE protocol (1.2 kV/cm × 100 µs × 8, 1 Hz).

For the CLS-354 cell line, the optimal concentration for bleomycin-based ECT was found to be 0.1 µg/mL (indicated by the vertical red dashed line). Increasing the BLM concentration beyond this point resulted in excessive cell death without PEF, which is sub-optimal for ECT.

### 2.3. Bleomycin-Based ECT in Combination with Nisin

**Figure 4:**
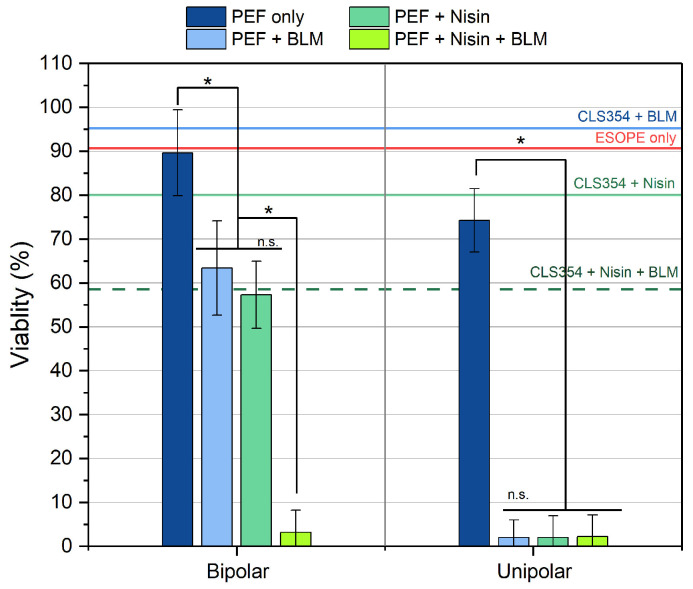
The dependence of cell viability on different treatment modalities (PEF only, nisin only, BLM only and various respective combinations). The green dashed line represents ECT efficacy with BLM and nisin without PEF. The cyan solid line shows cell viability when combined with nisin without PEF. The blue solid lines show ECT efficiency with BLM without PEF. The red dashed line represents average ECT efficiency using the ESOPE protocol (1.2 kV/cm × 100 μs × 8). Data presented as average ± SD. One-way ANOVA: * *p* < 0.05, n.s.—*p* > 0.05.

For the ECT experiments, cells were exposed to 150 pulses of 300 ns duration at 13 kV/cm, delivered at a frequency of 1.66 MHz. For bipolar bursts, symmetrical pulse sequences of ↑300 ns + ↓300 ns were applied. The BLM-based ECT was performed with and without nisin. After 48 h, the cytotoxic effects of the treatment were characterized (Figure 4).

The results showed that bipolar PEF (alone) did not significantly reduce cell viability, which was comparable to the viability observed after the ESOPE protocol (~91%), indicating predominantly reversible electroporation. In contrast, unipolar nsPEF reduced cell viability to ~75%, suggesting greater membrane permeabilization, which is in agreement with permeabilization data. BLM or nisin alone did not significantly affect cell viability (i.e., viability remained > 80%). However, the combined application of nisin and BLM reduced viability to 58%, indicating that nisin can be used as an adjuvant agent in BLM-based treatments.

The results, in combination with PEF, were of particular interest. In the PEF + nisin group, unipolar nsPEF bursts triggered a high, apparently saturated cytotoxic effect, suggesting that nisin may have potential as a standalone ECT agent. In the case of bipolar bursts, the effects of nisin were also evident; however, the BPC phenomenon still limited the treatment efficacy. The results are in agreement with the permeabilization data; however, a viability rate of ~63% is insufficient for ECT applications.

Finally, BLM-based ECT was evaluated with nisin used as an adjuvant agent. In all cases, the outcome was highly favourable, demonstrating saturated killing of all the cells, independent of the applied protocol that was triggered. These findings support the potential use of bipolar pulses in the ECT setting, achieving high cytotoxicity while potentially preserving the advantages associated with bipolar pulse delivery, i.e., reduced muscle contractions/pain, reduced oxidative effects and a more homogeneous treatment due to impedance mitigations.

## 3. Discussion

Our study demonstrates that nisin improves the efficacy of bleomycin-based ECT under both unipolar and bipolar high-frequency nsPEF conditions. Although symmetrical bipolar pulses are typically limited by BPC, our study reveals that nisin can mitigate this limitation and improve the treatment efficiency.

As BPC continues to pose a major limitation in bipolar-pulse ECT, several studies have sought to overcome or alleviate this effect. Foundational work showed that the opposite-polarity phase cancels nsPEF responses and that the cancellation weakens as the inter-phase delay increases—on the order of ~10 µs—underscoring timing as a key lever for mitigation [38].

Building on this, Valdez et al. [43] demonstrated that extending the second phase (e.g., +300/−900 ns) largely abolished cancellation and restored permeabilization to near-unipolar levels, whereas symmetric or short back-phases maintained strong BPC. In our recent work, a 0.1 ms interphase delay enabled saturated permeabilization at 10 Hz–1 kHz and enhanced cisplatin-based ECT [44]. Mechanistically, delaying the negative phase may allow for the partial recovery of membrane potential, thereby reducing cancellation effects. Longer delays (e.g., 10 ms at ~100 Hz) have been associated with transient hyperpolarization followed by depolarization during membrane relaxation [45]. Consistently, our previous work with Rembiałkowska et al. (2023) [42] demonstrated that MHz range bipolar pulse sequences with brief delays can approach unipolar efficiencies, albeit with greater total energy, particularly in calcium-based ECT. Notably, we also observed pronounced BPC for 500 ns pulses delivered at 1 MHz, both in vitro and in vivo [40]; maintaining efficacy at this frequency may require a substantial increase in the pulse amplitude. In the present study, the addition of nisin allowed for membrane permeabilization at 15 kV/cm across all tested frequencies, suggesting that nisin may help maintain electroporation efficiency under conditions where bipolar cancellation is typically pronounced. This confirms that the application of high-frequency bipolar PEF may require higher amplitudes to achieve high treatment efficiency. Together with prior reports [46,57], these findings support the view that bipolar cancellation is strongly dependent on pulse timing and frequency, with more pronounced effects at higher repetition rates (above ~1 kHz). Although the tested frequency range would typically be expected to promote bipolar cancellation under the applied conditions, in the present study, this effect was attenuated in the presence of nisin, resulting in sustained membrane permeabilization to YP across the examined protocols. Therefore, pulse repetition frequency alone does not independently determine membrane permeabilization efficiency.

At the same time, unipolar PEFs, when delivered at MHz frequency, offer superior efficiency of permeabilization when compared to the low-frequency protocols. Rapidly repeated pulses maintain a residual TMP throughout the burst [58]; thus, the interval between pulses may be so short that the membrane does not fully discharge to its resting potential before the next pulse arrives. This residual TMP is particularly beneficial, as it enables more efficient pore formation and allows for electroporation at lower field strengths [59,60]. This effect is evident in Figure 1, where the PEF protocol involved a pulse duration of 300 ns and a pulse repetition frequency of 1.66 MHz. Thus, an increase in the repetition frequency offers an additional means of controlling electroporation efficiency without the need to increase burst energy. However, during PEF procedures based on unipolar pulses, ion accumulation and electrolysis near the electrodes may increase local resistive heating when employed in vivo, whereas bipolar pulses reduce these effects by alternating polarity, preventing charge buildup and minimizing tissue or sample overheating [61]. Moreover, bipolar pulses have been reported in the literature to reduce net electrochemical reactions at the electrodes [62,63], reducing local pH shifts and thereby helping to preserve tissue integrity or medium stability. The higher frequency component of bipolar pulses is also suggested to contribute to more uniform field distribution and reduced impedance-related heterogeneity, resulting in a more homogenic treatment [64]. Therefore, bipolar pulses have clear advantages and significant potential for application in ECT; however, their use must be carefully optimized due to the presence of BPC, which can reduce electroporation efficiency and thus requires appropriate control or mitigation strategies. In this context, nisin may represent a promising adjunct, as it could help support membrane permeabilization under conditions where BPC is pronounced.

In our study, we also tried the 100 ns pulses; however, they were not successful. The result is influenced by Schwan’s equation, which implies that a certain time constant (τ) is required to charge the membrane sufficiently [64]. Therefore, higher amplitudes are needed to achieve the desired polarization and respective ECT effect with 100 ns pulses; however, it has restricted applicability in vivo due to the voltages involved. Even a 13 kV/cm PEF amplitude for a 1 cm tumour would require a 13 kV device. Further increases in the voltage in real applications pose safety and engineering challenges.

Nevertheless, it is evident that nisin can be successfully used in the context of ECT. Synergistic effects of PEF and nisin have been explored previously in bacteria with micro- and sub-microsecond pulses [55]. The rationale for using nisin to enhance ECT with symmetrical bipolar pulses stems from its ability to form stable pore complexes in cell membranes through Lipid II binding [65]. Yi et al. (2013) demonstrated that nisin lowers the threshold electric field required for liposomal content release, underscoring its potential as a pore-stabilizing agent [49]. We have also presented a proof-of-concept study recently, where we have shown that nisin can be used in ECT [66]. In this work, we wanted to weaken the BPC phenomenon, and the results suggest that this objective was achieved under the tested conditions. Our hypotheses and observed effects are in agreement with earlier reports suggesting that nisin contributes to pore stabilization, which is particularly important during bipolar cancellation. It should be noted that, while we were able to present a proof of concept in this study, further detailed research on the molecular interactions and changes in the membrane is required.

Previous studies on nisin-based anti-cancer treatments have demonstrated its potential in inhibiting cancer cell proliferation. Balcik-Ercin and Sever (2022) reported that nisin treatment of liver cancer cell lines reduced proliferation and triggered apoptosis at concentrations of 160 and 320 µg/mL, along with a decrease in drug resistance factors associated with cancer progression [67]. Norouzi et al. (2018) highlighted the anti-metastatic effects of nisin, demonstrating that concentrations of 40–50 IU/mL and 250–350 IU/mL suppressed the proliferation of colorectal cancer cell lines (LS180, SW48, HT29, and Caco2) [52]. Furthermore, Sadri et al. (2022) found that nisin exhibited lower cytotoxicity toward normal endothelial cells compared to cancer cell lines, with IC50 values ranging from 11.5 to 23 µM (38.5–77.1 µg/mL, respectively) [68]. Our study supports the beneficial effects of nisin in the cancer treatment context.

A limitation of this study is the use of a single cell line as the experimental model. Although this approach is appropriate for a proof-of-concept investigation and enables controlled evaluation of the proposed methodology, the biological responses observed may be cell type-specific. Differences in membrane composition, morphology, proliferation rate, and electroporation sensitivity across cell lines could influence treatment outcomes and PEF responses. Therefore, the present findings should be interpreted cautiously and not directly generalized to other cellular models or in vivo systems. Furthermore, although the enhanced effect observed in the presence of nisin may be associated with pore stabilization and interactions with Lipid II, the current study does not directly assess membrane dynamics or pore stability. Consequently, these mechanistic interpretations should be regarded as hypotheses supported by indirect evidence from Yo-Pro-1 uptake and viability measurements rather than definitive conclusions. Future studies should validate the proposed approach across multiple cell lines and more physiologically relevant models, as well as include direct mechanistic investigations to clarify the underlying membrane processes.

## 4. Materials and Methods

**Figure 5:**
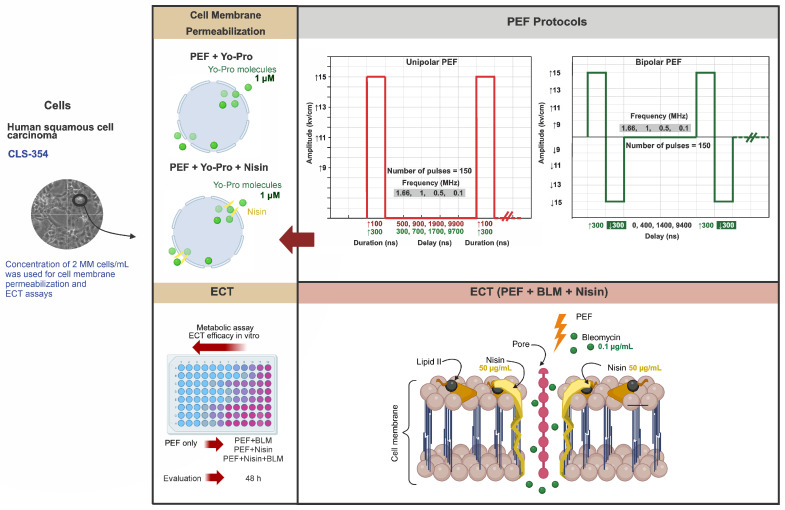
Experimental workflow for evaluation of nisin-assisted nanosecond electrochemotherapy in CLS-354 cells. Cells were treated with unipolar or bipolar nsPEFs (100 or 300 ns; 150 pulses) with or without nisin and bleomycin. Membrane permeabilization was determined by Yo-Pro-1 uptake, and metabolic activity was assessed 48 h post-treatment. ↑ and ↓ indicate positive and negative pulses, respectively. Red waveforms represent unipolar PEF pulses, while green waveforms represent bipolar PEF pulses.

This in vitro study investigated the combined effects of nisin (50 µg/mL) and ECT with bleomycin (0.1 µg/mL) on the human squamous cell carcinoma cell line CLS-354. The experiments evaluated the effects of unipolar and bipolar nanosecond pulsed electric fields with pulse durations of 100 ns and 300 ns on membrane permeabilization and cell survival. Bipolar pulses were delivered as symmetrical sequences of positive and negative pulses separated by short delays, allowing for the study of potential differences in cell-membrane permeabilization and biological response compared with unipolar pulses. The influence of pulse repetition frequency (0.1, 0.5, 1, and 1.66 MHz) on cell-membrane permeabilization and subsequent uptake of the green-fluorescent dye Yo-Pro-1 was assessed. In addition, cell viability under the different treatment conditions was determined. A simplified experimental scheme is shown in Figure 5.

### 4.1. Cells

Human squamous cell carcinoma CLS-354 cell line was obtained from (CLS—Cell Lines Service GmbH, Eppelheim, Germany). The cells were grown and maintained at 37 °C in 5% CO_2_ in DMEM with 4.5 g/L D-Glucose, 4 mM L-Glutamine, 3.7 g/L NaHCO_3_, and 1.0 mM Sodium pyruvate, supplemented with 10% fetal bovine serum, 100 U/mL penicillin, and 0.1 mg/mL streptomycin. On the experimental day, CLS-354 cells were detached using Trypsin-EDTA (Thermo Fisher Scientific, Waltham, MA, USA), then centrifuged and resuspended in electroporation buffer (10 mM HEPES, 250 mM Sucrose, 1 mM MgCl_2_) for further processing, depending on the experiment procedure. For permeabilization and metabolic viability assays, cells were resuspended at a concentration of 2 × 10^6^ cells/mL. Subsequently, cells were incubated on ice for 20 min, following the procedure for further assays. For cytotoxicity assays, cells were resuspended to a concentration of 5 × 10^4^ cells/mL. All cell culture reagents were sourced from Gibco (Thermo Fisher Scientific, Grand Island, NY, USA).

### 4.2. Preparation of Nisin

Nisin Z (>0.95% purity, Cas. No. 137061-46-2, Cat. No. 0305, Handary, Brussels, Belgium) was dissolved in sterile distilled water to prepare a 0.5 mg/mL stock solution. The final concentration of 50 µg/mL was selected, based on previous research [66], as the highest non-cytotoxic concentration. Before electroporation, cells were incubated with 50 µg/mL nisin for 20 min on ice. Following incubation, the cell suspension was transferred to an electroporation cuvette for pulse application.

### 4.3. Cell Permeabilization Detection Assay Using Yo-Pro-1

Cell permeabilization in CLS-354 cells triggered by electroporation was assessed using the green-fluorescent dye Yo-Pro1 (YP, Sigma-Aldrich, St. Louis, MO, USA). Cells suspended in electroporation buffer were mixed with YP to achieve a final concentration of 1 μM. A 50 μL aliquot of the mixed solution was placed between the electrodes and treated with various PEF protocols. The effects of bipolar pulses were also characterized with and without nisin (50 µg/mL).

**Figure 6:**
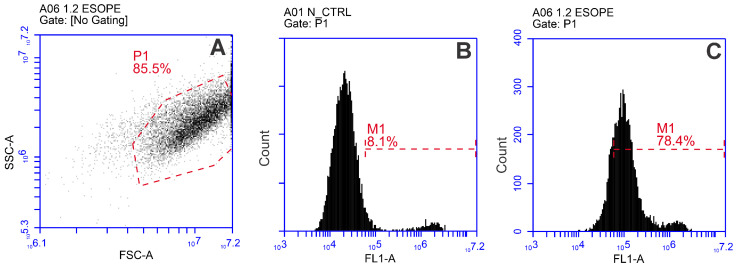
Gating strategy using flow cytometry: (**A**) singlet gate (FSC-A and SSC-A—forward scatter area and side scatter area, respectively), (**B**) example of gate definition (YP-positive cells, FL-1 channel) of negative/untreated control, (**C**) characteristic shift in spectra following electroporation (example of ESOPE protocol: 1.2 kV/cm × 100 μs × 8, 1 Hz).

After treatment, the cells were transferred to a 96-well round-bottom plate (Nunc, Sigma-Aldrich, St. Louis, MO, USA). Following 10 min incubation at room temperature, 150 μL of 0.9% NaCl solution was added to each well. The samples were then analyzed using a BD Accuri C6 flow cytometer (BD Biosciences, San Jose, CA, USA), where YP fluorescence (Ex. 491/509) was detected in Channel FL1 (Em. 533/30 nm BPF). The gating strategy used to determine YP-positive and unaffected cells is provided in Figure 6.

### 4.4. Concentration of Bleomycin for Electrochemotherapy

The most suitable concentration of bleomycin (BLM) to be used in ECT was assessed through the cytotoxicity experiments (without PEF). The cells (5  ×  10^4^/mL) were seeded on microscope cover slides in plates with or without BLM (Medac, Wedel, Germany) at varying concentrations (0–25 µg/mL). After 24 and 48 h of incubation, the slides were washed three times with phosphate-buffered saline (PBS) and stained with Trypan blue. The adherent cells on the slides were counted under a light microscope. Cell viability, determined by Trypan blue uptake, was found to be between 98.5% and 99.3%. The viability of cells after treatment was expressed as the percentage of remaining adherent cells relative to the control. For the ECT experiment, a range of BLM concentrations below IC50 was tested in the presence and absence of PEF (ESOPE, 1.2 kV/cm × 100 µs × 8) after 24 h and 48 h using the same procedure. A 0.1 µg/mL BLM concentration was selected as optimal for further ECT research.

### 4.5. Electroporation Setup and Parameters

Electric pulses were delivered using a high-voltage pulse generator capable of delivering biphasic pulses with durations ranging from 65 nanoseconds to 100 microseconds, at voltages of up to 3 kV [69]. The electroporation system employed a commercially available electroporation cuvette (Biorad, Hercules, CA, USA) with a 1 mm gap.

In the permeabilization assay, one hundred and fifty unipolar pulses with durations of 100 and 300 ns were applied at repetition frequencies ranging from 1.6 MHz to 0.1 MHz. The electric fields used in the experiment ranged from 9 kV/cm to 15 kV/cm, which were chosen to optimize the pulse lengths and ensure the effective permeabilization of the cell membrane through the application of pulsed electric fields (PEFs). Based on the data from experiments with unipolar pulses, the bipolar protocols were derived. Bipolar PEFs were delivered as symmetrical sequences of 300 ns pulses (↑300 ns + 0 ns + ↓300 ns + delay ns) using the same electric field amplitudes and pulse repetition frequencies as for unipolar exposures. The duration of delay was adjusted to achieve repetition frequencies of 0.1, 0.5, 1.0, and 1.66 MHz, corresponding to delays of 9400, 1400, 400, and 0 ns, respectively, whereas inter-phase delay was set to zero (maximal BPC). Based on the permeabilization results from both treatment modalities (unipolar/bipolar), the suitable protocols for ECT were derived.

As a result, protocols featured field strength of 13 kV/cm, selected as an optimal compromise between permeabilization efficiency and practical constraints related to electric field delivery, particularly considering the increased technical and safety limitations associated with higher field strengths in potential in vivo applications, delivering 150 pulses at a repetition frequency of 1.66 MHz, with pulse durations of ↑300 ns for unipolar and ↑300 ns + 0 ns + ↓300 ns + 0 ns for bipolar PEF modalities.

Additionally, the ESOPE protocol (1.2 kV/cm × 100 µs × 8, 1 Hz) served as a reference for successful ECT.

### 4.6. Statistical Analysis

We employed a one-way analysis of variance (ANOVA; *p* < 0.05) to compare the experimental groups. Differences were considered statistically significant at *p* < 0.05. When ANOVA indicated a significant effect, Tukey’s Honestly Significant Difference (HSD) multiple comparison test was applied to compare the differences between the groups. All data were processed using OriginPro software 2019b (OriginLab, Northampton, MA, USA). Each experiment was performed in at least three biological repetitions involving technical repetitions. Data are expressed as mean ± standard deviation.

## 5. Conclusions

Our study demonstrates that leveraging the synergistic action of nisin and PEF can attenuate bipolar cancellation (BPC) and verifies the applicability of bipolar pulses for ECT. Bipolar PEF protocols with 300 ns pulses showed minimal impact on permeabilization. The inclusion of nisin in these protocols significantly enhanced permeabilization, with the most notable effect seen at 15 and 13 kV/cm. The results showed that BLM-based ECT did not induce sufficient cytotoxicity with bipolar pulses due to BPC. However, the addition of nisin restored efficacy to a level comparable to that of ESOPE pulses, resulting in near-complete cell killing.

## Data Availability

The original contributions presented in this study are included in the article. Further inquiries can be directed to the corresponding authors.
